# Indoor Residual Spraying With Fludora Fusion for Malaria Control in Pyrethroid-Resistant Areas of Gujarat, India: A Community-Randomized Trial

**DOI:** 10.1155/jotm/9237780

**Published:** 2025-01-29

**Authors:** Raghavendra Kamaraju, Chandra Sekhar Pant, Sreehari Uragayala, Rajendra Kumar Baharia, Harish Chandra Srivastava, Rajpal Singh Yadav

**Affiliations:** ^1^ICMR-National Institute of Malaria Research, Sector 8, Dwarka, New Delhi, India; ^2^ICMR-National Institute of Malaria Research Field Unit, Civil Hospital, Nadiad, Gujarat, India; ^3^ICMR-National Institute of Malaria Research Field Unit, Poojanahalli, Bengaluru, India; ^4^Academy of Public Health Entomology, Udaipur 313002, India

**Keywords:** *Anopheles culicifacies*, bendiocarb, clothianidin, deltamethrin, indoor residual spray, vector control

## Abstract

**Background:** The development of insecticide resistance in malaria vectors has necessitated a need to evaluate new insecticide molecules with different modes of action. In the present study, Fludora Fusion 562.5 WP-SB (clothianidin 50% + deltamethrin 6.25% AI/kg) was evaluated for its efficacy and residual action for the control of pyrethroid-resistant malaria vector, *Anopheles culicifacies* (Diptera: Culicidae), during May 2017 to February 2018 in Gujarat state, India.

**Methods:** Fludora Fusion at the dose of 225 mg AI/m^2^ and bendiocarb at a dose of 400 mg AI/m^2^ as a positive control were sprayed in 5 villages each in districts of Kheda, Vadodara, and Panchmahal. The persistence of their efficacy on different local surfaces was determined against *An. culicifacies*. Entomological indices such as indoor resting density, human landing collections, pyrethrum spray collections, and exit trap collections were monitored to assess the impact of spraying.

**Results:** The observed residual action of Fludora Fusion on mud and cement surfaces was for 6 months and bendiocarb for 3–4 months on both surfaces. Indoor resting densities and parous rate of *An. culicifacies* were significantly lower in houses sprayed with Fludora Fusion compared to bendiocarb-sprayed houses. Daily entomological inoculation rate (EIR) declined from 1.275 during prespray period to 0.5225 in the Fludora Fusion arm and 0.3802 in the Ficam arm in postspray period, indicating a reduction in the malaria transmission potential of *An. culicifacies* in both arms.

**Conclusion:** Based on the residual action of the Fludora Fusion on most common sprayed surfaces and its effects on the elements of vectorial capacity, Fludora Fusion at 225 mg/m^2^ dose was found effective for more than 6 months and could be a potential option for the control of resistant mosquito vectors.

## 1. Introduction

Large-scale application of insecticides by indoor residual spraying has long been used to control malaria in India and other countries. Insecticide resistance in malaria vectors was reported to be a major impediment for the successful control of malaria in many countries where resistance to one or more classes of insecticides has been reported in malaria vectors. Due to the continuous spraying of insecticides in vector control programs the world over, malaria vector(s) have developed resistance to DDT and HCH (organochlorines), malathion (an organophosphate), and synthetic pyrethroids [1–5]. To prevent the onset of resistance, new insecticides may need to be developed with a mode of action that differs from those currently used in vector control programs [6]. These insecticides may also have to be combined to delay the development of resistance. Fludora Fusion is one such combination formulation developed by M/s Envu Environmental Science, U.S.A. (previously Bayer Crop Science, Germany). It consists of the active ingredient of clothianidin (500 g AI/kg; 50%) and deltamethrin (6.25 g Ai/kg; 6.25%) wettable powder packaged in water-soluble bags (WP-SB). Clothianidin is a neonicotinoid and widely used in agriculture, and its efficacy was evaluated in a few countries by indoor residual spraying where it was found to be effective in controlling malaria vectors [7–12]. Structurally, neonicotinoids resemble nicotine, which acts on the central nervous system of insects by stimulating nAChR and activating postsynaptic acetylcholine receptors, but not inhibiting AChE. It has been well established that neonicotinoids are highly effective, safe for mammals, have low toxicity, no cross-resistance, and have a particular mode of action and selectivity for insects, which may result from differences in the sensitivity of their receptors [13–19].

The two active ingredients of Fludora Fusion, i.e., clothianidin and deltamethrin, are intended to provide an effective solution for the disease control programs by addressing the challenge of insecticide resistance in malaria-transmitting mosquitoes [20]. The product has been found effective in field trials against different insecticide-resistant mosquito species when applied on a wide range of indoor surfaces [21]. In a study in India, indoor residual spray of clothianidin was found effective against *Anopheles culicifacies* (Diptera: Culicidae), the principal malaria vector in rural areas, for about 6 months [8, 9]. In other studies, in Benin [7], the Democratic Republic of the Congo [11], and Tanzania [12], clothianidin was found to be highly effective against both pyrethroid-susceptible and pyrethroid-resistant malaria vectors. In an experimental hut study in Benin, the combination of clothianidin and deltamethrin was shown to have residual action for 8–9 months (> 80% mortality in *An. gambiae* in cone bioassays) and demonstrated potential to control pyrethroid-resistant mosquito vectors and with prolonged duration of residual activity on sprayed surfaces [10].

In the present village-scale (Phase III) study, Fludora Fusion 562.5 WP-SB formulation applied as an indoor residual spray was evaluated for its efficacy on the malaria vector, *An. culicifacies* in comparison with bendiocarb 80 WP-SB as a positive control.

## 2. Methods

### 2.1. Test Items

#### 2.1.1. Fludora Fusion WP-SB

It is a WP-SB containing a combination of clothianidin (500 g AI/kg; 50%), a neonicotinoid, and deltamethrin (62.5 g AI/kg; 6.25%), a synthetic pyrethroid manufactured by Envu Environmental Science, U.S.A. It was applied at the target dose of clothianidin 200 mg AI/m^2^ and deltamethrin 25 mg AI/m^2^ (i.e., 225 mg AI/m^2^).

#### 2.1.2. Ficam 80 WP-SB

It is WP-SB containing bendiocarb (800 g AI/kg; 80%) and prequalified by the World Health Organization for IRS and manufactured by Envu Environmental Science, U.S.A. It was applied at the target dose of 400 mg AI/m^2^.

### 2.2. Study Area and Selection of Villages

**Figure 1:**
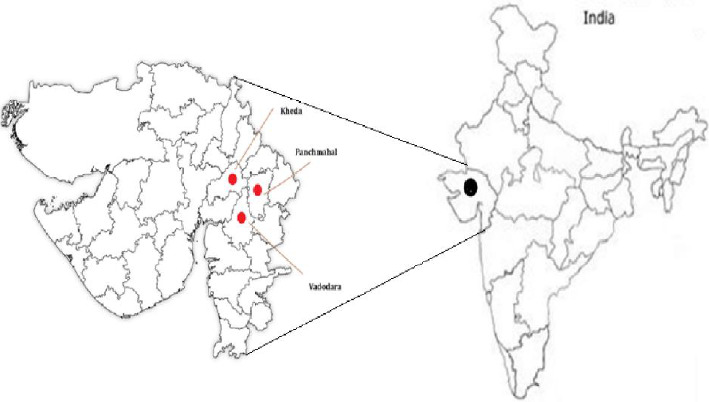
Map showing study sites in Kheda, Vadodara, and Panchmahal districts, Gujarat.

The baseline mosquito collections were done in 15 villages/hamlets of Kheda, Vadodara, and Panchmahal districts, and of them, 10 villages were selected for the studies based on adequate densities of *An. culicifacies* (Figure 1). These villages were located either on the bank of a perennial river or in the vicinity of an irrigation canal and are ∼1.5 km apart from each other. In the villages, houses were constructed of different wall materials, such as mud walls, mud/brick walls, cement brick walls, and roofs of corrugated tin sheets, earthen tiles, or cement concrete. Houses with earthen tile roofs generally have eaves that are 30 cm wide. Based on the baseline vector densities, villages were matched and randomly allocated to either Fludora Fusion WP-SB or Ficam WP-SB. Thus, each arm contained five villages where indoor residual spraying was applied. Entomological monitoring was carried out in three of the five villages of each arm to assess the impact of insecticides on the vectorial potential. Here, the term “arm” refers the two groups in the study: the Fludora Fusion group and the Ficam group.

### 2.3. Community and Information

The *Sarpanch* (elected head of the Village Council) and other members of the *Panchayats* (the Village Councils), staff of the health department, and other opinion leaders and villagers were explained about the purpose of the study, and their consent was obtained for their active cooperation. Information on the spraying was given to the Primary Health Centre (PHC) staff and villagers well in advance and again 3 days before the spraying operation commenced following the norms of the National Vector-Borne Disease Control Programme [22]. The spraying schedule was shared with the respective *Panchayats* and state health department in advance of the IRS and included details on the date of spray, population of the village, number of houses and rooms included in spray, name and quantity of insecticide, and numbers of squads to be deployed. Community consent was obtained in a transparent manner, ensuring that all participants understood the study procedures and their roles.

### 2.4. Indoor Residual Spraying

The monsoon season in Gujarat begins in the middle of June; malaria transmission takes place in the middle of July, so households in the trial villages were sprayed from late June to early July. The spraying was carried out with hand-operated compression sprayers (Hudson Xpert) equipped with a control flow valve (1.5 bar) and flat fan ceramic nozzle tips (8002E), in accordance with WHO guidelines [23, 24]. The spraying was carried out after pressuring the sprayer at 4 bar (58 psi) aiming for a discharge rate of 550 ± 10 mL/min or 30 mL/m^2^ surface area. Each spray pump was calibrated daily at the beginning of spray to ensure uniform quality of spraying to dispense the target dose on the sprayed surfaces. The spray applicators were given hands-on training on good spraying technique. One round of spray was applied in all accessible dwelling rooms and cattle shelters in the villages of both the arms, Fludora Fusion arm and Ficam arm.

The spraying accuracy on indoor surfaces was assessed in each of the two arms by selecting randomly one room on each side with mud walls, cement-plastered walls, and lime-plastered walls. Five Whatman No. 1 filter papers (circular with 15 cm diameter) were fixed at different wall heights (top, middle, and bottom of the walls) using steel pins ensuring some distance as a gap from the wall to avoid the contact of the sprayed filter paper with the wall surface. Thus, altogether, 50 filter papers were fixed on walls in each of the Fludora Fusion and Ficam sprayed arms before the spray and sprayed with insecticide.

The next morning, the dried filter papers were removed gently using steel forceps and packaged individually in aluminum foils. Samples of each surface and study arm were kept in plastic bags separately. Each foil and bag were coded and labeled with information such as date, village name, house ID, type of surface, insecticide, dose, and position of paper on the wall (top, middle, and bottom of the walls). The samples were stored in the refrigerator at +4°C–8°C temperature until they were sent to the WHO Collaborating Centre, Walloon Agriculture Research Centre, Gembloux, Belgium, for analysis of the contents of insecticides.

Before spraying, householders were advised to take safety precautions to avoid possible risks during and after the spray. They were asked not to scrub, damage, or plaster the walls until the end of the study. To assess any adverse effect of insecticides, all the spray men were interviewed using a questionnaire at the end of the day's spray, the morning after spraying, and 1 week postspraying. Six adult householders in each of the five villages sprayed with Fludora Fusion and four villages with Ficam were interviewed, respectively, after 1 and 4 weeks after completion of the spray round.

### 2.5. Susceptibility of *An. culicifacies* to Insecticides

The susceptibility of wild-caught F_1_ females of *An. culicifacies* and a laboratory colony mosquito to deltamethrin (0.05%) and bendiocarb (0.1%) were determined according to the standard WHO method [25, 26] before and after the spray (July 2016 and October 2017).

### 2.6. Assessment of Residual Efficacy and Persistence

The efficacy and persistence of insecticidal action on the sprayed surfaces were determined using standard WHO cones following the WHO guidelines [27]. For bioassays, locations to fix cones on sprayed surfaces were marked one day after the spray. The cone bioassays were carried out on mud-plastered and cemented wall surfaces sprayed with Fludora Fusion or Ficam on days 7 and 15 postspray and then every month in each study village. Control surfaces (unsprayed controls) were selected in the unsprayed houses in the same village.

The cones were fixed on the surfaces using adhesive tape. Laboratory-reared, 3–5 days old, sugar-fed females of *An. culicifacies* (deltamethrin-resistant strain) were used in the bioassays. Five replicates of 10 *An. culicifacies* females were tested for each insecticide and the surface type. Mosquitoes were gently removed from the cones after 30 min of exposure and transferred to paper cups covered with netting. During the study, mosquitoes were provided access to cotton wool saturated with a 10% sugar solution and were kept in a laboratory maintained at 27°C ± 2°C and 75 ± 10% RH. Upon exposure, knockdown was observed at one hour, and mortality was observed at 24, 48, and 72 h following exposure. The bioassays were carried out until the corrected mortality remained ≥ 80%. When the corrected mortality fell below 80% at 72 h postexposure in the bioassays on two consecutive occasions, the tests were terminated and repeated after 15 days to confirm the results.

### 2.7. Impact of Spraying on Vector Population

To measure the impact of spraying, key entomological parameters were monitored, and data were recorded on a fortnightly basis except for human landing collections and light trap collections that were done at monthly intervals following standard entomological techniques [28].

For this, 4–5 suitable mud-plastered houses were selected in three villages in each of the two trial arms based on the baseline vector densities. Additionally, two suitable cement-plastered houses in these villages were also selected for conducting cone bioassays. For entomological monitoring, the rooms were suitably modified by covering the eaves from inside by green/black cotton cloth hung like a curtain funnel from the top frame allowing mosquito entry through the eave. Before pyrethrum spray collection (PSC), the eaves were closed by pulling a cord/string tied with the lower part of the cloth to prevent mosquito exit. In every sentinel monitoring house, two exit window traps were fitted on the windows/walls with an iron frame facing the north/east direction allowing early morning light. Informed consent of the heads of all households selected was obtained using a consent form in the local language (Gujarati). The following entomological collections were made for assessing different parameters.

#### 2.7.1. Floor Sheet Collections

These collections were done fortnightly for recording overnight mortality of mosquitoes in sprayed rooms. White cloth sheets were spread out on the entire floor of the room in the evening before the households retired to bed. The next morning, the dead and morbid mosquitoes lying on the floor sheet were collected with forceps and placed in cups with moistened cotton wool, transported to the laboratory, identified to species, and recorded. Other dead insects lying on the floor were collected separately and stored for monitoring. Precautions were taken to protect the knocked-down mosquitoes from scavengers such as ants.

#### 2.7.2. Exit Trap Collections

A cleaning of the exit traps was conducted in the designated houses prior to sunset with the assistance of the householders. After opening the exit trap the next morning, live mosquitoes were collected using an aspirator, transferred to plastic cups covered with netting, and provided with cotton wool pads soaked in a 10% glucose solution. After holding them for 24, 48, and 72 h, delayed mortality was recorded. In order to identify the species of mosquitoes, dead mosquitoes were carefully collected from the exit trap and placed in cups containing moist cotton wool.

#### 2.7.3. Hand Collections and PSCs

These collections were done fortnightly. A mosquito collector in each room collected resting mosquitoes (indoors) in the house using an aspirator and flashlight for 15 min following the floor sheet and exit trap collections. The live mosquitoes were kept in cups, labeled, and provided with cotton wool pad moistened with 10% sugar solution, and the cups were placed in a laboratory maintained at 27°C ± 2°C and 75 ± 10% RH to observe delayed mortality after 24, 48, and 72 h.

Pyrethrum solution (0.2% pyrethrum extract in kerosene) was sprayed using a hand sprayer starting from the inner corner of the room and exiting the room subsequently. The room was opened and ventilated well after 15 min. The mosquitoes found dead on the floor sheet were picked up with forceps. Species of mosquitoes were identified, and gonotrophic conditions of females were noted.

#### 2.7.4. Light Trap Catches

Light trap collections were done at monthly intervals in one sentinel village in each arm. Two battery-operated CDC miniature light traps were hung 1.5 m above the floor (one indoor + one outdoor) in one village of each arm and operated from 18:00 to 06:00 h under the supervision of a staff. The next morning, the trapped mosquitoes were collected, identified to species, and the gonotrophic condition of females was recorded. The vector species specimens were preserved for the sporozoite ELISA test.

#### 2.7.5. Human Landing Collections

Dusk-to-dawn human landing catches (indoor and outdoor) were made in one sentinel village of each arm at monthly intervals, following the standard WHO method [28]. The landing mosquitoes were collected from the local bait volunteers (one each at outdoor and indoor places) throughout the night from 18:00 to 06:00 h. Informed consent of each volunteer was obtained before their inclusion in the study. In order to collect mosquitoes for blood feeding, the collectors took care to collect mosquitoes from their exposed legs before probing for blood. Each hour of the night, the number of mosquito species caught was recorded. The ovaries of female vectors were extracted in the field laboratory to determine the parous rate, and their heads and thoraces were preserved for the sporozoite ELISA test.

#### 2.7.6. Measurement of Sporozoite Rate, Parous Rate, and Human Blood Index

Mosquitoes collected by different methods from sentinel houses were identified and categorized by species, as well as their gonotrophic condition. Blood of freshly fed *An. culicifacies* was smeared on Whatman No. 1 filter paper, and after drying, the filter papers were stored appropriately for the determination of the human blood index (HBI) of *An. culicifacies* by the gel diffusion technique [29]. The heads and thoraces or whole specimens of *An. culicifacies* were preserved on silica gel crystals in the Eppendorf tubes for the sporozoite ELISA test [30–32]. Specific monoclonal antibodies were used to identify *Plasmodium vivax* and *Plasmodium falciparum*. The parous rate of unfed *An. culicifacies* female was determined by checking ovarian tracheolar skeins by dissection of abdomen [28].

Measurement of entomological inoculation rate (EIR) and human biting rate: The EIR is calculated by multiplying the sporozoite rate and the human landing rate to obtain the number of infective bites per person per unit of time [33]. A human biting rate was calculated by calculating the number of bites per person per night (b/p/n) based on human landing collections.

The information on mosquitoes collected was processed in the following order:

• Number of vector mosquitoes found dead on floor sheet in the morning……… (a)
• Number caught resting in the room (hand collection)……… (b)
• Number found dead on floor after pyrethrum spray……… (c)
• Number found dead + alive in exit traps……… (d)
• Number found fed in exit traps……… (e)

Comparisons between treatments and control were made, and the inferences were drawn as under:

• Immediate mortality = a + nos. dead in exit trap
• Indoor resting density = b + c
• Total mosquito entry rate = a + b + c + d per room per night……… (f)
• Excito-repellency = exit to entry rate (d/f) × 100
• Proportion of fed to total entry in treatment and control……… (g)
• Proportion of fed to gravid in treatment and control

Delayed mortality = Number dead after 24/48/72 h among the live mosquitoes caught in room and trap/total entry × 100.

Overall mortality = (Number dead on floor + trap+ 24/48/72 h holding)/f × 100.

Success in feeding……… g.

Parity = proportion of parous to nulliparous………… p.

### 2.8. Data Entry and Analysis

**Figure 2:**
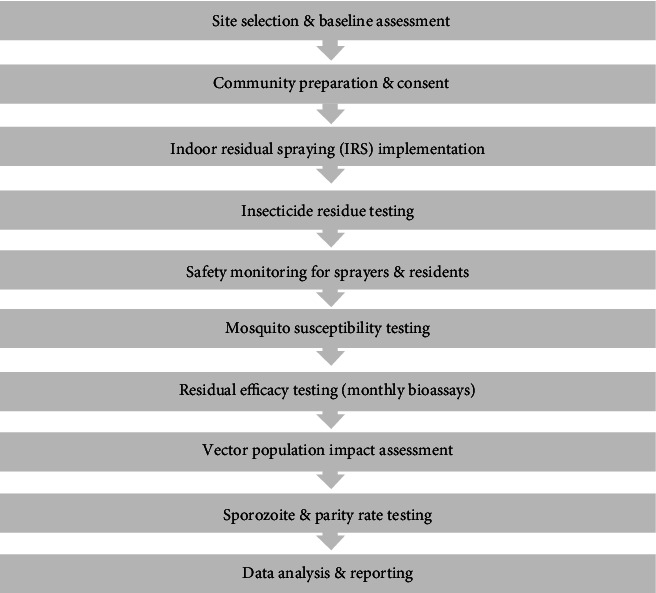
Flowchart outlining the key methodology steps.

Data were entered in MS Excel sheets. Generalized linear mixed models with negative binomial log link were used to find the difference in mortality proportion between the two trial arms and surfaces. Separate models were created for 24-, 48-, and 72-h mortality. Mosquito densities were analyzed statistically using generalized negative binomial regression, and geometric mean densities were presented. The Fisher exact test was used for proportionate variables to see the differences between the two arms. Delayed mortality in mosquitoes collected from sprayed houses was assessed using binomial regression. The outline of the method key steps is mentioned in Figure 2.

The entomological parameters were calculated as follows:

1. Total mosquito density (entry) = total number of mosquito species caught indoors by hand catch and pyrethrum spray + total number of mosquitoes collected on spray sheets + number found in exit traps
2. Induced exophily: proportion of mosquitoes exiting the room: calculated as number caught in exit traps/total number of mosquitoes entered the room
3. Mortality: calculated as total number dead = (immediate + delayed mortality)/total number of mosquitoes collected

## 3. Results

The susceptibility tests showed that the wild-caught and the colonized strains of *An. culicifacies* were resistant to deltamethrin (mortality, 68.3%–75%) but susceptible to bendiocarb (mortality–100%).

The numbers of houses and rooms sprayed in the study villages are shown in Table 1. In the Fludora Fusion arm, altogether 703 houses (2084 rooms; 3144 population) were targeted for spray. The house coverage rate was 89.76%, and the room coverage was 89.35%. In the Ficam arm, 749 houses (2328 rooms; population 3273) were sprayed. The spray coverage of houses was 90.79%, and the room coverage rate was 91.97%.

Based on the chemical contents of filter paper samples collected from sprayed rooms, Table 2 shows the results. The mean applied-to-target ratio of deltamethrin content in Fludora Fusion sprayed houses was 0.45 on mud-plastered walls and 0.4 on the lime-coated cement-plastered surfaces and that of clothianidin was 0.51 on mud surfaces and 0.46 on lime-coated cemented walls. In the Ficam arm, the applied-to-target ratio of bendiocarb was 0.59 on mud-plastered walls and 0.69 on cement-plastered wall surfaces. The results showed the application of lower than targeted dosages in both arms, which may be attributed to application errors.

**Figure 3:**
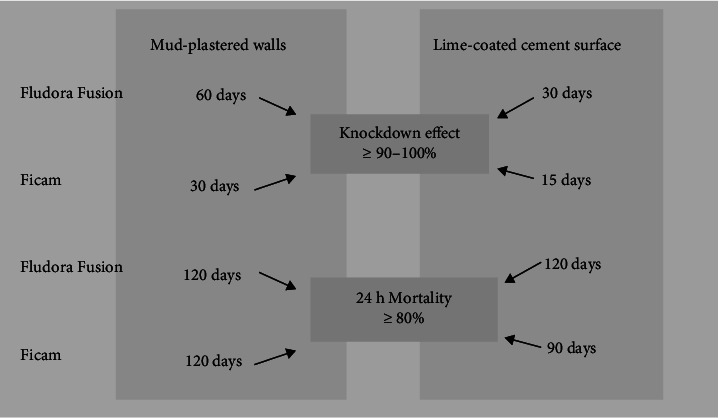
Insecticidal activity of Fludora Fusion and Ficam on mud and lime-coated cement surfaces showing knockdown and mortality over time.

An assessment of the insecticidal activity was made by cone bioassay tests on common surfaces, i.e., mud-plastered walls and lime-coated cement surfaces sprayed with Fludora Fusion and Ficam formulations until 8 months postspraying (Table 3). On mud-plastered walls sprayed with Fludora Fusion, 1-h knockdown was ≥ 90%–100% up to 60 days, whereas on lime-coated cement surface, it was ≥ 90%–100% for 30 days. The surfaces sprayed with Ficam (bendiocarb) formulation, the 1-h knockdown effect was ≥ 90%–100% for 30 days on mud and lime-coated cemented surface effective for 15 days. Fludora Fusion WP-SB sprayed on mud and lime-coated cement surface caused ≥ 80% up to 120 days on cement-plastered walls and 180 days on mud-plastered walls. The 24-h mortality (≥ 80%) of *An. culicifacies* on mud and lime-coated cement surface sprayed with Ficam was up to 120 days and 90 days, respectively (Figure 3).

Considering a cutoff value (knockdown effect below 90% and residual effect below 80%), it was observed that Fludora Fusion formulation was more effective in knocking down mosquitoes than Ficam formulation on different surfaces. On each surface, the effect of Fludora Fusion formulation persisted longer than the Ficam. It was observed that extended holding of mosquitoes exposed on different surfaces sprayed with Fludora Fusion and Ficam, i.e., for 48 h and 72 h made marginal increase in the mosquito mortality for both formulations (Table 3).

The highest mortality was recorded within 24 h postexposure. There was a significant difference in mosquito mortalities between the surfaces for both the insecticides at 24 h (*χ*^2^ = 6.5; *p*=0.01), 48 h (*χ*^2^ = 6.917; *p*=0.009), and 72 h (*χ*^2^ = 7.226; *p*=0.007) holding postexposure. Furthermore, there was no significant difference between the mortalities of both insecticides at 24-h holding period (*χ*^2^ = 2.59; *p*=0.107), whereas there was a significant difference at 48-h (*χ*^2^ = 4.2; *p*=0.040) and 72-h (*χ*^2^ = 4.54; *p*=0.033) holding periods, indicating that Fludora Fusion (clothianidin + deltamethrin) was on par or more efficacious than the Ficam (bendiocarb) when the holding period was extended.

### 3.1. Effect of Spraying on Mosquitoes

In all, 3317 *Anopheles* spp., 731 *Culex* spp., and 49 *Aedes* females were caught in different methods from the study villages during the study. The proportion of the malaria vector, *An. culicifacies*, was 41.15% of all anopheline species. *Anopheles stephensi* and *An. fluviatilis* were found in low numbers. The former species is mainly an urban malaria vector. In Gujarat state, *An. fluviatilis* sibling species T alone has so far been found, which is a nonvector species among the members of the complex. *Anopheles annularis* is a malaria vector of secondary importance elsewhere in eastern India. Thus, in this study, the effectiveness of Fludora Fusion and Ficam was considered relevant for reduction of the vectorial potential of *An. culicifacies*.

**Figure 4:**
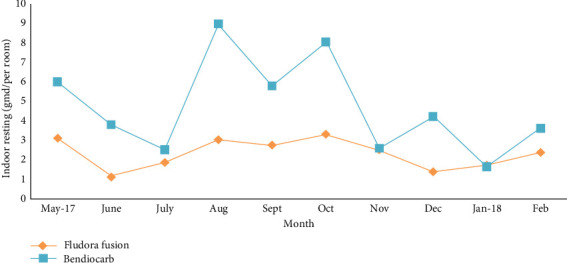
Geometric mean indoor resting density of *An. culicifacies* in villages sprayed with Fludora Fusion WP-SB or Ficam WP-SB formulations.

The indoor resting population density of *An. culicifacies* varied owing to the extrinsic factors in the baseline period (May to June 2017) in both trial arms, the geometric mean densities were nearly comparable in both arms of the trial, and there was no significant difference between the arms for *An. culicifacies* density and that of other mosquitoes (*p* > 0.05). After the spraying in late June 2027, the density of *An. culicifacies* declined in villages of both arms in July 2017. Thereafter, there was sharp increase in the density in villages sprayed with Ficam (bendiocarb) from August to September. Overall, the density was lower in villages of Fludora Fusion arm as compared to Ficam arm until the end of the trial (Figure 4). During the 3 months (August to October 2017) postspraying, the results of contact bioassays on sprayed walls showed mortality in *An. culicifacies* of ≥ 80% in both the arms, and the geometric mean densities remained lower in the Fludora Fusion sprayed villages than those in the Ficam sprayed villages (*p* < 0.003). This also indicates intrinsic slow-acting nature of the Fludora Fusion.

The mean parous rate of *An. culicifacies* in villages of both the arms during the baseline period ranged from 49.09% (14/30) to 42.44% (37/69), and these rates did not differ significantly (Fisher's exact test, *p* > 0.05). During the initial 3 months after spraying (August to October), the parous rate in both arms declined gradually. However, the reduction in parous rate in villages sprayed with Fludora Fusion (144/682) was lower as compared to the villages of the Ficam arm (98/360), and there was a significant difference between the arms in the postspray period (Fisher's exact test, *p* < 0.05).

During the baseline period, blood meal samples of *An. culicifacies* collected from villages were pooled for analysis, and the HBI of *An. culicifacies* was 4.76% (8/168). The numbers of blood meals reactive to the antisera of both humans and animals were combined to estimate the HBI. During postspraying, 426 and 288 blood meal samples of mosquitoes were collected from Fludora Fusion and Ficam arms, respectively. The HBI in the Fludora Fusion and Ficam arms was 3.05% (13/428) and 3.0 (8/266), respectively. HBI did not differ significantly between arms (*p* > 0.05; Fisher's exact test).

Data on EIR using mosquitoes collected off human baits and light trap, pyrethrum spray, and exit trap collections are given in Table 4. The presence of sporozoites of *P. falciparum* and *P. vivax* (strain 210 and 247) was recorded by ELISA. During the baseline prespray period, the sporozoite rate was 0.866% (5/577) and the daily EIR rate was 1.275%. In the postspray period, the sporozoite rate was 0.220% (2/909) in the Fludora Fusion and 0.338 (2/591) in the Ficam arm. After spraying, the daily EIR declined to 0.5225 in the Fludora Fusion arm and 0.3802 in the Ficam arm, indicating a reduction in the malaria transmission potential of *An. culicifacies* in both arms.

**Figure 5:**
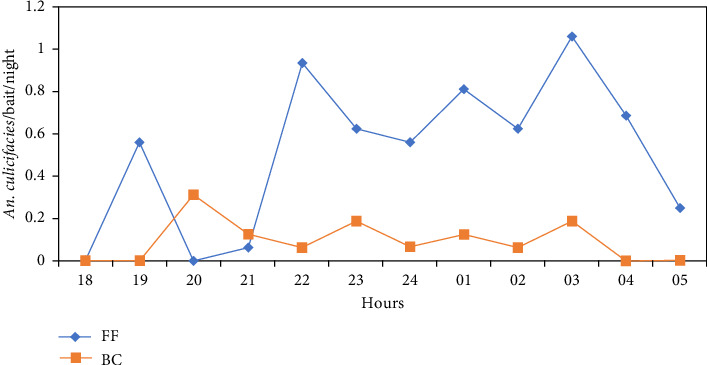
Number of female *An. culicifacies* caught landing per human bait volunteer per night in both the study arms.

Details of the number of mosquitoes landed per night per bait are shown in Figure 5. The data showed that more *An. culicifacies* mosquitoes landed on the human baits in Fludora Fusion sprayed houses than the Ficam sprayed houses throughout the study period, indicating indirectly a low excito-repellent property of clothianidin.

The proportion of fed to total entry and number of fed to gravid of *An. culicifacies* in sprayed rooms are given in Table 5. There was a marginal difference in the proportion of fed to total entry of mosquitoes between the two arms of the trial. Since HBI was very low in both areas (3.051%–3.007%), it appears that many female mosquitoes were entering human dwellings for resting after biting animals. The proportions of fed to gravid in Fludora Fusion arm were higher as compared to the Ficam arm. These parameters indicate that Fludora Fusion formulation had a low excito-repellency effect, thus allowing a longer exposure of mosquitoes resting on the sprayed surfaces, thereby resulting in mortality of the female mosquitoes from reaching to semigravid/gravid stages. Of the total number of females that entered the rooms, the exit rate did not vary significantly in the two trial arms. The excito-repellent effect in both arms was nearly on par. The data indicate that both the insecticide formulations had the least excito-repellent action.

The number of mosquitoes collected per light trap per night in the Ficam sprayed houses was lower than those from the Fludora Fusion sprayed houses indicating a comparatively lower repellency of clothianidin. *An. stephensi* mosquitoes were collected in very low numbers.

### 3.2. Perception of the Spray Men and Householders on Possible Adverse Effects

#### 3.2.1. Householders

A total of 109 and 72 persons were interviewed from Fludora Fusion and Ficam arms, respectively. None of them reported any discomfort or adverse effects 1 week and 1 month after spraying their houses.

#### 3.2.2. Spray Men

None of the 10 spray men interviewed at the end of the working day, the next morning, and after a week reported any discomfort or adverse effects.

## 4. Discussion

A randomized village-scale trial of Fludora Fusion 562.5 WP-SB versus Ficam 80 WP-SB sprayed indoors at 225 mg AI/m^2^ and 400 mg AI/m^2^ dose, respectively, was carried out in Gujarat State during May 2017 to February 2018 for the control of pyrethroid-resistant *An. culicifacies*, a key vector of malaria in this area. Results of the bioassays on mud-plastered and lime-coated cement-plastered walls, which were the most common surfaces in the study villages, were found that Fludora Fusion caused ≥ 80% mortality of *An. culicifacies* up to 6 months on mud-plastered surfaces and up to 4 months on lime-coated cement-plastered surfaces, respectively, while Ficam caused ≥ 80% mortality of *An. culicifacies* up to 3 months on mud surfaces and 4 months on lime-coated cement walls, respectively, when the holding period was 24 h postexposure. However, an extended holding period up to 72 h postexposure increased the residual action of Fludora Fusion by 2 months on lime-coated surface. The indoor resting density of *An. culicifacies* was lower throughout the study period in villages sprayed with Fludora Fusion as compared to Ficam. The decline in parous rate in Fludora Fusion arm was higher. Analysis of the proportion of fed to total entry and fed to gravid *An. culicifacies* in sprayed rooms indicated that Fludora Fusion formulation had a low excito-repellent action as compared to Ficam, allowing a longer exposure of mosquitoes resting on the Fludora Fusion sprayed surfaces, resulted in mortality of the female mosquitoes approaching the semigravid/gravid stages.

In a study conducted by Gueye et al. [34] against An. coluzzii and An. arabiensis reported 11 months efficacy of Fludora Fusion with the laboratory strain and 2 months residual efficacy with the wild strain, however the study reported increased efficacy with increased holding periods for (72 and 96 h). In the present study, extended holding period showed 2 months more efficacy period than the efficacy obtained with 24-h holding. Thiomela et al. [21] in experimental hut studies in Cameroon also reported the high efficacy of Fludora Fusion indoor residual spray. Similarly, Fongnikin et al. [35] reported 7–10 months efficacy of Fludora Fusion indoor residual spray in Benin, Africa. A study in Ethiopia by Animut and Horstmann [36] also reported that the efficacy of Fludora Fusion in controlling resistant malaria vectors. Fuseini et al. [37] reported efficacy of > 80% after 72-h exposure for a period of 8 months in Equatorial Guinea and stated that the combination formulation of Fludora Fusion was quite effective under field conditions.

In general, reduction in the elements of vectorial capacity, i.e., vector densities, parous rate, and survivorship of female *An. culicifacies*, was greater in villages sprayed with Fludora Fusion 562.5 WP-SB during the trial period than in the villages sprayed with Ficam 80 WP-SB. Daily EIR declined from 1.275 during prespray period to 0.5225 in the Fludora Fusion arm and 0.3802 in the Ficam arm in postspray period, indicating a reduction in the malaria transmission potential of *An. culicifacies* in both arms. There was no adverse reaction reported by sprayers or residents during spraying or afterward. Considering these results, Fludora Fusion applied at the dose of 225 mg/m^2^ is effective for 6 months on different sprayed surfaces available in the villages of Gujarat. The efficacy would be longer if the applied dose of the products was within ±25% of the target doses, which is the acceptable tolerance limit. Based on the present study results and studies conducted elsewhere, the Fludora Fusion combination formulation with clothianidin and deltamethrin could be a potential option for indoor residual spray in controlling malaria vectors that are reporting resistance to different classes of insecticides including pyrethroid. In areas with seasonal malaria transmission, one round of indoor residual spray may be opted for control of malaria vectors to reduce the cost of repeated spray operations.

Insecticide resistance management is a major issue worldwide. In India, pyrethroid resistance is a major concern to overcome the resistance issue. In a previous small-scale Phase II trial, the residual efficacy of Fludora Fusion WP-SB (using the WHO cutoff mortality of ≥ 80%) persisted above 120 days (4 months) postspraying on both mud-plastered walls and lime-painted walls [38]. Similarly, in the present study, the large-scale Phase III trial, the observed residual action of Fludora Fusion on mud and cement surfaces was for 180 days (6 months), and bendiocarb for 3–4 months on both surfaces. In spray surface, Anopheles density and parous rate found lower. Fludora Fusion Phase III trial has been found suitable at the area where synthetic pyrethroid found resistant.

## 5. Conclusion

Based on the residual action of the Fludora Fusion on most common sprayed surfaces and its effects on the elements of vectorial capacity, Fludora Fusion at 225 mg/m^2^ dose was found effective for more than 6 months and could be a potential option for the control of resistant mosquito vectors.

## Figures and Tables

**Table 1:** Study villages and coverage of human dwellings and rooms under spraying in both arms of study.

District	Village	PHC	Population	Targeted		Sprayed		Percent	
				House	Room	House	Room	House	Room
*Fludora Fusion*(225*mg*/*m*^2^)									
Panchmahal	Bhamrana Muvada	Timba	793	161	538	137	447	85.09	83.09
	Gothada	Timba	697	146	477	134	436	91.78	91.40

Vadodara	Varsada	Vejpur	623	167	460	146	408	87.43	88.70

Kheda	Shakti Nagar	Alina	513	111	259	100	235	90.09	90.73
	Umba	Nes	518	118	350	114	336	96.61	96.00

		Total	3144	703	2084	631	1862	89.76	89.35

*Ficam*(400*mg·AI*/*m*^2^)									
Panchmahal	Dharoda	Timba	565	133	481	125	453	93.98	94.18

Kheda	Navi Nagari	Sevaliya	546	115	248	91	213	79.13	85.89
	Aklacha	Nes	739	191	591	176	548	92.15	92.72
	Bhadrasa	Nes	862	182	590	168	533	92.31	90.34
	Hatipura	Salun	561	128	418	120	394	93.75	94.26

		Total	3273	749	2328	680	2141	90.79	91.97

**Table 2:** Chemical analysis of samples of Fludora Fusion (clothianidin + deltamethrin) and Ficam.

Insecticide	Type of surface	No. of samples	Mean content mg/m^2^ (range)	Applied-to-target dose ratio⁣^∗^	No. of samples in the acceptable range (1 ± 0.5)
*Fludora Fusion*					
Clothianidin	Mud-plastered walls	25	102 (15–261)	0.51	9
	Lime-coated cement walls	25	91 (< 10–285)	0.46	8

Deltamethrin	Mud walls	25	11.2 (1.7–25.4)	0.45	8
	Lime-coated cement walls	24	10.0 (1.4–29.5)	0.4	6

*Ficam*					
Bendiocarb	Mud walls	25	235 (91–522)	0.59	11
	Lime-coated cement walls	24	266 (48–889)	0.69	7

⁣^∗^The optimal applied-to-target dose ratio is ±25%.

**Table 3:** Duration of persistence of effectiveness (in days) of formulations sprayed on different surfaces.

Surface	Effect	Fludora Fusion 562.5 WP-SB	Ficam 80 WP-SB
Mud-plastered walls	Knockdown⁣^∗^	60	30
	24-h mortality⁣^∗∗^	180	90
	48-h mortality⁣^∗∗^	180	120
	72-h mortality⁣^∗∗^	180	120

Lime-coated cement-plastered surfaces	Knockdown⁣^∗^	30	15
	24-h mortality⁣^∗∗^	120	120
	48-h mortality⁣^∗∗^	180	120
	72-h mortality⁣^∗∗^	180	120

⁣^∗^≥ 90%.

⁣^∗∗^≥ 80%.

**Table 4:** Entomological inoculation rate in study arms.

Arms	Period	Samples assayed	Assay results			Sporozoite rate (%)	Biting rate	EIR %
			Pf	Pv 210	Total			
Baseline	Prespray	577	4	1	5	0.87	1.5	1.275

Fludora Fusion	Postspray	909	0	2	2	0.22	2.5	0.5225
Ficam		591	1	1	2	0.338	1.28	0.3802

Abbreviations: Pf, P. *falciparum*; Pv, *P. vivax*.

**Table 5:** Entry and excito-repellency and indoor resting of *An. culicifacies* females in villages in the postspray period.

Insecticide sprayed	Postspray period								
	Entry (f)	Dead + alive in exit trap (d)	Fed in exit trap (e)	Fed	Gravid	Fed/entry	Fed/gravid	Exit/entry (d/f)	Fed in trap/entry (e/f)
Fludora Fusion	555	2	0	312	136	0.560	0.245	0.004	0
Ficam	644	6	1	251	106	0.389	0.165	0.009	0.002

## Data Availability

Data are contained within the article.
